# Annotations

**Published:** 1908-02-08

**Authors:** 


### ANNOTATIONS.

### Kissing the Book.

A recent case, carried on appeal from the Bloomsbury County Court to the Divisional Court, presents a somewhat novel aspect of the discussion relating to the method of taking the oath by witnesses in a court of law. Among the witnesses at the first trial was a medical man, who brought with him a copy of the New Testament, and on this he wished to be sworn. The judge, however, refused to allow the proposal, and suggested that if the witness objected on sanitary grounds to the court Testament he could take the oath in the Scottish fashion. But the witness expressed his disapproval of this method, and as he also refused to subscribe the oath in the usual manner, the judge would not permit him to give evidence. During the hearing of the appeal this action of the County Court judge was made the subject of complaint, and Mr. Justice Phillimore remarked on it in the course of his judgment. While allowing that it was necessary to exercise some care in reference to a book brought to court by the witness himself, on the ground that it might not be a complete copy of the Testament, he expressed the opinion that the judge would have been wiser, in the case under discussion, to have looked at the Testament in order to satisfy himself that it was a genuine copy—which, presumably, he refused to do. We have frequently entered a protest against the dirty and risky practice of kissing a book soiled by hands and lips of all and sundry, and have advised that medical witnesses should help to popularise a more sanitary practice by accepting the option now allowed by the law of swearing with uplifted hand. It now seems that to this there may be, in the minds of some, objections or scruples, and witnesses who entertain these may find a way of escape in the method sanctioned by Mr. Justice Phillimore.

### Syphilis in the Third Generation.

In the course of his recent address to the Society for the Study of Disease in Children, Mr. R. Clement Lucas referred to the much-discussed question whether inherited syphilis is capable of transmission to the third generation. On the negative side he quoted from his own practice a case having beyond question very impressive features. Both the parents were blind from interstitial corneo-iritis, and they both presented other typical evidences of inherited disease—namely, notched upper central incisors and scars at the angles of the mouth. Yet the child which was the issue of this marriage remained in good health and free from all evidences of specific taint during the months it was kept under observation. Striking as this instance is, it is, however, far from being conclusive. In the first place it is purely a negative observation, and thus its force is a limited one. Secondly, it must be remembered that, because in the earlier months or years of life no evidence of syphilis appears, this is by no means a guarantee that this freedom will continue; such a condition as interstitial keratitis, for example, may be delayed until perhaps the second or third decade. Hence Mr. Lucas's case, as, of course, he recognises, though interesting and important, is far from proving that even in the individual instance in question syphilis was not transmitted to the third generation; and still less does it establish a general negative on the point at issue. When examples in the opposite direction are quoted, there is always the possibility that one or other of the parents, in addition to the inherited taint, had also suffered from acquired syphilis, and that what looks like transmission to the third generation is merely an ordinary example of congenital specific disease. Hence it seems impossible to say with absolute confidence either "Aye" or "No" to the question whether a parent, the subject of inherited, and not of acquired syphilis, may transmit the disease to his offspring. But it may certainly be stated that such transmission, if it occurs at all, is quite unusual and exceptional.

### Subcutaneous Fibrous Nodules.

The recognition and description of small subcutaneous fibrous nodules as a feature in the clinical expression of rheumatism in early life, dates from the clasical paper of Barlow and Warner read at the London meeting of the International Medical Congress in 1881. It is now universally allowed that the conclusions expressed by these authors were substantially correct, though experience has produced some modification in respect to detail. The original claim included the proposition that the nodules were indicative of rheumatism, even in the absence of pain, and also that they had no origin or connection other than rheumatism. Concerning the latter of these statements, it has since been conclusively shown that subcutaneous fibrous nodules do occasionally occur in rheumatoid arthritis. It is true that no clinical confusion between the two groups of cases is probable, for the latter disease mainly declares itself in adults, and when subcutaneous nodules accompany it they are usually much larger and more persistent than those which are so characteristic a note of rheumatism in childhood. But cases intermediate between the two extremes just stated undoubtedly occur, and thus, as a matter of doctrine, it is impossible any longer to maintain the proposition that subcutaneous fibrous nodules are a conclusive proof of rheumatism unless at the same time it is admitted that rheumatoid arthritis falls within this category; and this latter view is actively controverted by most writers on the subject. A good illustration of the doctrinal difficulties here presented is afforded by a recent case shown to the Medical Society by Dr. Parkes Weber. The patient, an adult male, had numerous large nodules in the neighbourhood of each elbow joint, but, in addition, there was also present on the back of one of the fingers a fibrous nodule of the size of a small pea—that is, in a patient with evidences of rheumatoid disease, nodule formation, in one instance at least, simulated the characters commonly seen in the rheumatism of childhood. It is therefore manifest that the question of size does not afford an absolute distinction between the nodules of rheumatism and those of rheumatoid arthritis. Not only is such a distinction a mere matter of degree, but there are actual instances in which the rule in regard to the size of the nodules is set at defiance.

